# Wie kann eine assistierte Technologie pflegende Angehörige im Pflegealltag unterstützen? Ergebnisse einer Machbarkeitsstudie zur App Liv

**DOI:** 10.1007/s16024-022-00371-5

**Published:** 2022-06-01

**Authors:** Eva Jansen, Annett Horn, Wolfram Herrmann

**Affiliations:** 1 Fachbereich Gesundheit, Fachhochschule Münster, Leonardo-Campus 8, 48149 Münster, Deutschland; 2 Professur für Allgemeinmedizin mit Schwerpunkt Versorgungsforschung, Charitéplatz 1, 10117 Berlin, Deutschland

**Keywords:** Technische Unterstützungsmöglichkeit, Digitale Gesundheitskompetenz, Pflege zu Hause, Digitale Unterstützung, Demenz, Technical support solution, Digital health literacy, Care at home, Digital support, Dementia

## Abstract

**Hintergrund:**

Aufgrund der älter werdenden Bevölkerung und der Zunahme von Menschen mit chronischen Erkrankungen im Alter, steigt die Zahl der pflegenden Angehörigen sowie deren Wert für die Versorgung. Dabei agieren sie meist ohne Vorerfahrungen im Bereich der Versorgung, haben begrenztes Verständnis für das Gesundheitssystem und erfüllen die Aufgabe neben der Bewältigung des eigenen Alltags. Daher gewinnen technische Unterstützungsmöglichkeiten wie die App Liv (entwickelt im Projekt münster.land.leben) an Bedeutung. Ziel der App Liv ist die Verbesserung der digitalen Gesundheitskompetenz der vulnerablen Gruppe der pflegenden Angehörigen in Deutschland.

**Ziel der Arbeit (Fragestellung):**

Ziel dieser Studie war, einen Prototyp der App Liv partizipativ mit pflegenden Angehörigen zu testen und das Konzept der App weiterzuentwickeln.

**Material und Methoden:**

Mit 10 pflegenden Angehörigen wurden unter Zuhilfenahme eines Prototyps der App qualitative online Interviews geführt, mittels qualitativer Inhaltsanalyse ausgewertet.

**Ergebnisse:**

Die pflegenden Angehörigen bewerteten das Layout und die Einfachheit der Funktionen insgesamt positiv. Als Voraussetzung für die Nutzung betonten die Interviewten die Einfachheit der Anwendung und eine Vielfalt an Dokumentationsmöglichkeiten. Erweiterungsvorschläge erfolgten in den Bereichen Spezifizierung der Aktivitäten, Hinzufügung von weiteren Kommunikationsformen, Reminder oder Bestätigungen für erledigte oder zu erledigende Aufgaben, Verweis auf weiterführende Informationen, Verknüpfung mit anderen digitalen Systemen und Hinterlegung wichtiger Dokumente.

**Diskussion:**

Aus Sicht der Betroffenen stößt die App auf eine Versorgungslücke. Auf Grundlage der Vorschläge der Interviewteilnehmerinnen werden 6 Erweiterungen für die App diskutiert. Um die App nicht zu überfrachten, müssen diese in einer weiteren Studie auf ihre Nützlichkeit getestet werden.

## Abstract (en)

**Background:**

Because of the constantly aging population and the number of people suffering from chronic illnesses in old age, the number of family caregivers and their role in care provision is increasing. Most of these people are acting without any prior experience in the care sector, have a limited understanding of the healthcare system and carry out the tasks alongside the demands of their own day to day lives. Technical support solutions such as the Liv app (developed in the project münster.land.leben) are therefore gaining traction.

The objective of the Liv app is to improve the digital healthcare expertise of the vulnerable group of family caregivers in Germany.

**Objective of the paper (research question):**

The goal of this study was to test the prototype of the Liv app and refine the concept with the participation of family caregivers.

**Material and methods:**

Qualitative online interviews were conducted with 10 family caregivers with the aid of a prototype of the app and evaluated using a structured, qualitative content analysis.

**Results:**

The family caregivers rated the layout and the simplicity of the features as positive overall. As a condition for them to use the app, the interviewees stressed its ease of use and the quantity of documentation provided. Suggestions for enhancements were related to specifying activities, including additional forms of communication, reminders and confirmation, providing links to more information, integrating the app into other digital systems and enabling important documents to be saved.

**Discussion:**

From the perspective of those affected, the app meets a huge unserved need. Based on the interviewees’ suggestions six enhancements to the app are under discussion. To avoid cluttering up the app, their usefulness needs to be tested in another study.

## Hintergrund

Pflegende Angehörige stellen eine tragende Säule der Versorgung von chronisch erkrankten und pflegebedürftigen Menschen dar. Das wird sich auch in den kommenden Jahren nicht ändern, denn wie Prognosen zeigen, wird die Zahl pflegebedürftiger Menschen weiter stetig ansteigen (Kuhlmey et al. 2014).

Zugleich ist zu beobachten, dass die Zahl derjenigen, die Pflege beruflich ausüben, weiter abnimmt. Demnach werden zukünftig fast 500.000 Vollzeitkräfte in der Pflege fehlen. Es entsteht eine zunehmende Lücke zwischen den Personen, die Pflege benötigen, und der Anzahl qualifizierter Pflegekräfte (Ehrlich und Kelle 2019; Rothgang et al. 2009).

Insbesondere in der häuslichen Versorgung – dort, wo die meisten pflegebedürftigen Menschen leben – fällt häufig die gesamte Verantwortung auf Familienangehörige und dabei meist auf eine einzelne Person zurück. Sie kümmert sich meist ohne Vorerfahrung als Hauptpflegeperson rund um die Uhr um die Gesundheit und Versorgung nahestehender pflegebedürftiger Menschen und muss zusätzlich ihren eigenen (Arbeits‑)Alltag weiterhin aufrechterhalten (Bohnet-Joschko 2020).

Dabei bleibt wenig Zeit für eigene Bedürfnisse. Nicht selten führt diese Situation zur Dauerbelastung und in der Folge leiden Angehörige unter Burn-out, Depressionen oder anderen (psychischen und physischen) Erkrankungen (Müller 2020; Rutz et al. 2019a; Wetzstein et al. 2015; Zhang et al. 2016). Hinzu kommen der Mangel eines Unterstützungsnetzwerks für pflegende Angehörige, unzureichende Gesundheitskompetenz und eine häufig damit einhergehende geringe Inanspruchnahme bestehender Unterstützungsstrukturen (Kurz und Wilz 2011; Metin et al. 2019).

In der Vergangenheit wurden wiederholt Versuche unternommen, pflegende Angehörige mit präventiven und gesundheitsförderlichen Maßnahmen, wie z. B. der Umsetzung von Ausgleichs- und Rückenübungen oder anderen Methoden, physischer und psychischer Überlastung vorzubeugen (Stiftung ZQP 2022), in ihrem Alltag zu unterstützen. Jedoch belegen Studien auch, dass pflegende Angehörige eine schwer zu erreichende Zielgruppe sind, da sie Angebote aus Zeitgründen, und weil sie keine Ersatzpflegeperson finden, oftmals gar nicht oder nur selten wahrnehmen (Lichte et al. 2018).

Es liegt daher nahe, digitalisierte Angebote für diese Zielgruppe zu entwickeln. Insbesondere Technologien auf dem Smartphone sind für Interventionen im Gesundheitswesen geeignet, da sie sowohl mobile Telekommunikation, Benachrichtigungen sowie die Möglichkeit zur Integration von klinischen Anwendungen beinhalten können und niederschwellig zu erreichen sind (Mainz und Zündel 2017). Studien zeigen, dass mobile Technologien ein wirksamer Ansatz zur Überwachung der Gesundheit für Menschen mit unterschiedlichen chronischen Erkrankungen sein können (Hardinge et al. 2015; Maier-Rigaud und Böning 2020).

In den letzten Jahren entwickelten sich zahlreiche assistierte Technologien für pflegebedürftige Menschen, wie z. B. Apps zur Verbesserung der eigenen kognitiven Leistungsfähigkeit für Demenzkranke (z. B. Zeuner und Rettinger 2019). Vergleichsweise wenig ist jedoch in Bezug auf die Lebenssituation pflegender Angehöriger passiert (Brown et al. 2019; Parker et al. 2008), obwohl der Gebrauch einer assistierten Technologie große Chancen zur Verbesserung der Gesundheitskompetenz birgt (Franke et al. 2019). Diese Lücke möchte die App Liv schließen.^1^

Die App besteht aus 4 Hauptfunktionen:

- Mit dem *Planer* und einer Erinnerungsfunktion lassen sich Aufgaben und Termine rund um den Pflegealltag organisieren und mit anderen Mitgliedern teilen.
- Der zweite Bereich *Pflege* bietet eine Übersicht des Gesundheitszustandes der zu pflegenden Person, inklusive der Dokumentation von relevanten Vitalfunktionen.
- Der dritte Bereich *Unterstützung* bietet durch Übermittlung der Informationen und Aufgaben die Möglichkeit, die Pflege temporär an andere Liv-Mitglieder abzugeben.
- Der vierte Bereich *Profil* ergänzt die App mit Angaben zur Hauptpflegeperson.

## Zielsetzung und Forschungsfrage

Ziele der hier beschriebenen Untersuchung waren eine partizipative Testung der App-Funktionen und Weiterentwicklung des Konzeptes mit Vertreter:innen der Zielgruppe. Geklärt werden sollte:

- für welche pflegenden Angehörige die App infrage kommt,
- welche Erwartungen pflegende Angehörige an eine digitale Unterstützung haben,
- wie die Zielgruppe die App-Idee auf Inhalte und Handhabbarkeit bewertet, und
- ob Inhalte fehlen oder ersetzt werden müssen.

Die so gewonnenen Erkenntnisse sollten dazu beitragen, den App-Prototyp zu vervollständigen und die Inhalte der App den Bedürfnissen der Zielgruppe anzupassen.

Als Zielgruppe wurden pflegende Angehörige zwischen 40 und 60 Jahren, die in der Region Münster leben und eine pflegebedürftige Person im häuslichen Pflegealltag versorgen, avisiert.

## Methode

Ursprünglich war die Studie als 3 Fokusgruppeninterviews mit integriertem Usability Test zu je 6 Teilnehmenden geplant. Aufgrund von Einschränkungen im Rahmen der COVID-19-Pandemie und Terminschwierigkeiten fanden schlussendlich Einzelinterviews mit 10 pflegenden Angehörigen statt. Diese wurden über eine Kontaktaufnahme zu ambulanten Pflegediensten, Selbsthilfegruppen und Vereinen in und um Münster, Anzeigen in Netzwerk-Apps für pflegende Angehörige, Foren und Facebook Gruppen rekrutiert und erhielten 50 € Entschädigung. Die Darstellung des App-Prototyps wurde über die Programme Flinto und Zoom realisiert.

### Erhebungsinstrumente

Die Einzelinterviews fanden zwischen dem 16.12.2020 und dem 04.02.2021 statt. Die leitfadengestützten Interviews wurden mit Einverständnis der Teilnehmenden aufgezeichnet und im Anschluss transkribiert. Der Leitfaden umfasste insgesamt 10 semistrukturierte Fragen zur allgemeinen Situation mit der zu pflegenden Person, zur App und zu ihren Funktionen. Der allgemeine Teil wird in diesem Artikel nicht wiedergegeben. Beim zweiten Teil erklärte und demonstrierte die Interviewerin die App; gleichzeitig hatten die Teilnehmerinnen die Möglichkeit, selbst einige Funktionen über eine Fernsteuerung auszuprobieren. Dies nahm eine Teilnehmerin wahr. Anschließend wurden die Teilnehmenden mithilfe des Leitfadens befragt.

### Auswertung

Die Auswertung erfolgte in Anlehnung an die qualitative Inhaltsanalyse (Kuckartz 2018; Mayring und Gahleitner 2019) mithilfe des Programms F4analyse. Aus dem anonymisierten Material wurden induktiv Codes bestimmt, strukturiert und zu Kategorien zusammengefasst. Gelenkt von den ursprünglichen Forschungsfragen erfolgte zunächst eine intensive Sichtung des Materials. Als Nächstes wurden Auswertungskategorien am Material und ein Kodierleitfaden entwickelt, modifiziert und ergänzt. Zuletzt erfolgte die Erstellung von einer quantifizierenden Materialübersicht in Form von Tabellen mit Häufigkeitsangaben zu den Auswertungskategorien.

## Ergebnisse

### Sample

Bei den 10 Teilnehmerinnen der Interviews handelt es sich ausschließlich um Frauen im Alter von 26 bis 58 Jahren (im Schnitt 48,6 Jahre). Mit Ausnahme eines zu pflegenden Sohnes liegt bei allen anderen zu pflegenden Personen eine diagnostizierte Demenzerkrankung mit einem Pflegegrad zwischen 2 und 5 vor. Sechs der pflegenden Angehörigen haben keinerlei regelmäßige medizinische oder nichtmedizinische Dokumentation durchzuführen. Die durchschnittliche Dauer einer Interviewaufnahme betrug 44:01 min. Eine Einzelauflistung der Teilnehmerinnen findet sich in Tab. 1.

**Table 1:** 

Pflegende Angehörige	Liv01	Liv02	Liv03	Liv04	Liv05	Liv06	Liv07	Liv08	Liv09	Liv10
*Alter und Geschlecht*	40, w	58, w	26, w	54, w	53, w	53, w	49, w	57, w	50, w	46, w
*Pflegebedürftige/Pflegebedürftiger*	Sohn	Mutter	Großmutter	Schwiegermutter	Mutter	Mutter	Mutter, Schwiegervater	Schwiegervater und Schwiegermutter	Vater	Vater
*Alter und Geschlecht*	14, m	86, w	85, w	88, w	81, w	81, w	80, w; 88 m	78–90, m; 84–90, w	81, m	77, m
*Diagnose*	ADHS und Autismus	Demenz	Demenz, Diabetes, Herzkrankheit	Demenz, Arthrose	Alzheimer-Demenz	Demenz	Demenz	Demenz	Alzheimer-Demenz	Alzheimer-Demenz
*Pflegegrad*	2	4	3	2	5	4	4, 3	4, 4	3	3
*Pflegesituation*	Zu Hause und Sonderschule	Zu Hause und Tagespflege	Zu Hause	Zu Hause	Beim Ehemann und Tagespflege	Zu Hause und Tagespflege	Mutter zu Hause (Tagespflege) Schwiegervater zu Hause	Im Heim und zu Hause	Im Heim und zu Hause	Zu Hause, Tagespflege
*Interviewdauer in Minuten*	53:24	52:48	28:15	29:42	45:39	34:54	40:01	81:52	35:17	38:20

### Kategorien

Die Codes ließen sich 6 Kategorien zuordnen: Herausforderungen der Pflege vor der Pandemie, zusätzliche Herausforderungen in der Pflege aufgrund der Pandemie, Voraussetzungen für die Nutzung der App, Erweiterungsvorschläge für die App, positive Gesamtbeurteilung der App und negative Gesamtbeurteilung der App. Die 4 App-bezogenen Kategorien werden im Folgenden im Detail vorgestellt.

### Voraussetzungen für die Nutzung

Aus Sicht der Teilnehmerinnen war die Möglichkeit, die App zusammen mit anderen an der Pflege beteiligten Personen zu nutzen, die wichtigste Voraussetzung (Tab. 2). Eine Frau berichtet hierzu: „Wenn ich jetzt alleine wäre, ich brauchte das nicht. (…) Da würden mir jetzt zwei Personen einfallen, ne drei, die könnten diese App nutzen und wären auch bereit, das zu erlernen. Bei einer Person wäre das ein Selbstläufer, bei meiner Schwester, die überhaupt nicht affin ist, mit Technik, der müsste ich das erstmal schön verkaufen, und bei meiner Tante müsste ich es bewerben“ (Liv02).

**Table 2:** 

Voraussetzungen für die Nutzung
Beteiligung der anderen Pflegenden an der Nutzung (6)
Zuständigkeit und Quantität der Dokumentation (4)
Funktionalität der Einweisung und des Einspeisens (2)

Anzahl der Nennungen in Klammer

Die Dokumentation medizinischer Werte in der App spielte aus Sicht der Interviewpartnerinnen keine große Rolle. Die Hälfte der befragten pflegenden Angehörigen gab an, dass sie bei ihren pflegenden Angehörigen keine medizinisch relevanten Werte regelmäßig zu dokumentieren haben. Auch waren sie der Ansicht, dass die App nur in vollem Umfang genutzt werden kann, wenn ein Austausch über medizinisch relevante Werte in der täglichen Pflege zentral ist. So sagte eine Teilnehmerin: „Diese Werte brauch ich nicht. Die hat sonst nichts. Die hat sonst keine Begleiterkrankungen, kein Diabetes und kein Bluthochdruck“ (Liv05). Weiterhin betonten zwei Teilnehmerinnen die Wichtigkeit, einer einfachen und angeleiteten Handhabung bei der Erstinstallation der App.

### Erweiterungsvorschläge

Als Erweiterungsvorschlag wurde am häufigsten die Möglichkeit einer Spezifizierung der Aktivitäten, die im Planer aufgelistet werden, genannt (Tab. 3). Zum einen betrifft dies eine qualitative Erweiterung: Dazu gehört, z. B. statt des einfachen Items „Mittagessen“ detaillierter angeben zu können, was die zu pflegende Person gegessen hat. Eine Teilnehmerin bemerkte: „Oder, dass er jetzt bei mir doch schon wieder was Süßes gegessen hat und so weiter, dass man das dann einträgt. Das finde ich super“ (Liv09).

**Table 3:** 

Erweiterungsvorschläge
Spezifizierung der Aktivitäten (7)
Weitere Kommunikationsformen (4)
Reminder oder Bestätigung (3)
Verweis auf weiterführende Informationen (3)
Verknüpfung mit anderen digitalen Systemen (2)
Hinterlegung wichtiger Dokumente (2)
Bereitstellung von weiteren Versionen (2)
Übermittlungsmöglichkeiten der Daten (2)
Hinzufügung einer allgemeinen To-do-Liste (1)
Personalisierung der Farben (1)

Anzahl der Nennungen in Klammer

Weiterhin wünschten sich einige Teilnehmerinnen eine grundsätzliche Auflistung von Vorlieben und Abneigungen gegenüber Speisen, um anderen betreuenden Personen die Pflege und Bewirtung zu erleichtern.

Drei Teilnehmerinnen schlugen vor, ein Trinkprotokoll mit genauer Angabe der Trinkmenge in dem Planer unterzubringen, sodass bei einem Betreuungswechsel klar ersichtlich ist, ob ein Mangel besteht. Das Gleiche gilt auch für die Menge an Nahrungsmitteln, die die zu pflegende Person zu sich nimmt. Eine Teilnehmerin formulierte es so: „Das Frühstück ist hinterlegt, aber kann man auch eintragen, wie viel sie gegessen hat? Und was auch wichtig wäre, ist ein Trinkprotokoll. Gerade Ältere trinken wirklich wenig“ (Liv03). Auch die Kontrolle der Anzahl der Toilettengänge war für einige pflegende Angehörige von großer Bedeutung.

Hilfreich wäre aus Sicht der Teilnehmerinnen darüber hinaus ein detailliertes Sturzprotokoll, das Muster im Sturzverhalten erkennt und über den Planer einzugeben ist.

Ebenfalls häufig gewünscht wurde eine Erweiterung der möglichen Kommunikationsformen, um den Austausch zwischen den an der Pflege beteiligten Personen zu erleichtern. Die Teilnehmerinnen wünschten sich insbesondere die Möglichkeit, Rundmails zu schreiben, und die Option, auf kurzem Wege schnell Nachrichten zu schreiben, z. B. über einen Chat. Ein Beispiel für den Nutzen einer Rundmailfunktion beschrieb diese Teilnehmerin: „Dass man hier Pflegegrad 3 oder 4 drin hat, und dass dann alle Bescheid wissen, wenn sich was ändert und was dann bedeutet. Diese Info müsste dann an alle rauszuschreiben sein“ (Liv09).

Bei vielen Teilnehmerinnen bestand eine Unsicherheit darin, ob die anderen, an der Pflege beteiligten Personen, alle Aufgaben ihren eigenen Vorstellungen entsprechend meistern. Oft handelte es sich bei Letzteren um ihre Kinder, um die Angestellten der Tagespflege oder auch um bezahlte Helferinnen. Um nicht ständig aktiv kontrollieren zu müssen, wünschten sie sich eine Reminderfunktion, die weitere an der Pflege Beteiligte automatisch an wichtige Aufgaben erinnert und die dann durch Abhaken als erledigt markiert werden können. Eine Teilnehmerin stellte sich das folgendermaßen vor: „Und dann noch mal ein Haken mit ‚bin bei ihm‘. Das wäre für mich eine Unterstützung, dann muss ich nicht jedes Mal anrufen, auch bei meinem Bruder. Das muss ich jedes Mal machen, weil ich Angst habe, dass sie es doch vergessen“ (Liv10).

Ob die App weiterführende Informationen bereitstellen soll, die die Pflege direkt, ihre Finanzierung oder ihr Management betreffen, darüber waren sich die Teilnehmerinnen nicht einig. Drei der Teilnehmerinnen hielten dies für sehr sinnvoll, 2 andere waren jedoch der Meinung, dass die App dadurch überladen werden könnte. Eine Teilnehmerin wünschte sich konkrete Informationen in der App: „Ich weiß nicht, ob in der App irgendwas vorgesehen ist, so ein Glossar mit Links, was mache ich, wenn z. B. einen neuen Rollstuhl brauche oder andere Hilfsmittel. (…) Nur wenn ich z. B. heute google Pflegedienst [*Ort*], dann kriege ich von Google 27.000 Mitteilungen, ich hätte aber eigentlich nur gerne eine Liste mit 5 oder 6 Adressen mit Telefonnummern und vielleicht auch mit der Aussage: nimmt zur Zeit keine neuen Patienten“ (Liv08). Andere Pflegende wünschten sich Links zu interessanten Artikeln oder zu einem Pflegewiki.

Teilnehmerinnen, die bereits mit digitalen Messgeräten (z. B. Blutzucker) oder Kalendern (z. B. Outlook) arbeiten, erhofften sich, diese mit der App einfach verbinden zu können, um die Daten nicht mehrfach einspeisen zu müssen. Von 2 Teilnehmerinnen wurde vorgeschlagen, wichtige Dokumente wie Vollmachten, Patientenverfügungen oder Impfpässe in der App hinterlegen zu können. Dies sei besonders für weitere an der Pflege Beteiligte interessant und hilfreich, falls ein Notfall in Abwesenheit der Hauptpflegeperson auftritt. Zwei Teilnehmerinnen fänden die gebündelte Übertragungsmöglichkeit – digital per E‑Mail oder analog in Form von Ausdrucken – von medizinisch relevanten Werten oder eines Sturzprotokolls an medizinisches Personal wie Ärzt:innen oder den Notdienst als vorteilhaft für ihren Alltag. Eine pflegende Angehörige wünschte sich eine Version der App, die von Pflegebedürftigen selbst ebenfalls genutzt werden kann. Eine andere Teilnehmerin stellte sich eine weitere Version für technisch weniger versierte Mitbetreuende vor, die ausschließlich mitlesen sollten. Zwei weitere Vorschläge betrafen das Hinzufügen einer allgemeinen To-do-Liste und die Personalisierung durch Farben in der Kalenderfunktion, um damit zu kennzeichnen, welche der zusätzlich mitbetreuenden Personen die entsprechend Aufgabe übernommen hatte.

### Gesamtbeurteilung

Die Mehrheit – 6 von 10 Teilnehmerinnen – beurteilte die App insgesamt sehr gut (Tab. 4). Sie äußerte sich dazu größtenteils in einem Kommentar, der sowohl die Einfachheit der Unterfunktionen, die Übersicht als auch das angenehme Layout adressierte. So sagte eine Teilnehmerin beispielsweise: „Find ich gut, es ist dezent, das find ich gut. Ich finde es auch klar und nicht zu viel, weil jetzt, wenn ihr noch mehr da reinpackt, dann wird es irgendwann zu viel, aber so sind es nur 4 Hauptsachen, und mehr ist nicht. Zu viel würde ich nicht reintun, dann wird es wieder zu kompliziert für mich persönlich, ich bin dann da immer überfordert. Aber das ist klar. Das ist auch von der Grafik her wirklich deutlich. Und selbsterklärend“ (Liv10). Acht von 10 befragten pflegenden Angehörigen gaben auch an, die App sofort nutzen zu wollen. Von den Teilnehmerinnen wurde das Nachrichtenübermittlungssystem der App jedoch als unpersönlich wahrgenommen: „Da schätze ich dann auch das Gespräch, dieser Smalltalk, den man dann auch etwas minimieren könnte. Ich würde mit meinen Schwestern nicht nur Werte austauschen, sondern auch, wie ist die (Pflegeperson) drauf“ (Liv07). Eine Teilnehmerin gab an, dass ein großer Teil der App, nämlich der Wertebereich, bereits von anderen, sich regulär auf Smartphones befindenden Apps abgedeckt wird (z. B. Apple Health).

**Table 4:** 

Gesamtbeurteilung positiv	Gesamtbeurteilung negativ
Ansprechendes Design und geringe Komplexität (6)	Unpersönlichkeit des Austausches (2) Nutzlosigkeit bestimmter Funktionen (1)

Anzahl der Nennungen in Klammer

## Diskussion

Diese qualitative Interviewstudie zeigt, dass sich eine Pflege-App wie Liv aus Angehörigenperspektive v. a. für pflegende Angehörige eignet, die die sich mit weiteren Personen um ihre zu pflegenden Angehörigen kümmern und die technisch versiert sind. Bei einer hohen Notwendigkeit der Dokumentation von medizinischen Werten bekommt eine solche App aus Angehörigenperspektive eine zusätzliche Relevanz. Darüber hinaus ergab die Angehörigenperspektive 6 Erweiterungsoptionen für die App:

Im Vordergrund steht die *Personalisierung der Planerfunktion*, sodass Nutzer:innen sie mit qualitativem Inhalt füllen können (zu Mahlzeiten, Trinkmenge, Vorlieben oder Abneigungen). Dies spielt insbesondere dann eine Rolle, wenn sich die zu pflegende Person nicht mehr selbst dazu äußern kann.

Die zweite Erweiterung betrifft die *Kommunikationsformen* in der App. Die Möglichkeit von Rundmails oder Chatfunktionen in der Kommunikation mit anderen pflegenden Angehörigen war von grundlegender Wichtigkeit in den Interviews. Dazu gehören auch Reminder- und Kontrollfunktion für Aufgaben, die andere übernehmen (wie z. B. in Brown et al. 2016 oder Garfield et al. 2016). Die Möglichkeit, kurze, selbst aufgenommene Videos als Kommunikationsform zwischen den pflegenden Angehörigen kursieren zu lassen, wurde in einer App aus den USA als erfolgreich befunden (Davis et al. 2014) und könnte für die Erweiterung dieser App sinnvoll sein.

*Ein Informationsbereich* als dritte mögliche Erweiterung in der App könnte über eine Baumstruktur kurz und knapp Antworten zu Fragestellungen anbieten, die sich in unmittelbaren Pflegesituationen ergeben. Diese sind in den allgemeinen Medien zwar ebenfalls zugänglich, diese informieren jedoch oft zu umfangreich oder müssen vorab mühselig gefiltert und selektiert werden (hierzu auch Horn et al. 2021). Das Bedürfnis, erwünschte Informationen in wenigen Schritten einfach zur Verfügung stehen zu haben, gilt für Smartphone-Nutzer:innen und besonders für technikaffine pflegende Angehörige, da diese über sehr wenig Zeit verfügen (Klawunn et al. 2021; Price et al. 2020; Queluz et al. 2020).

Eine zusätzliche Website birgt die Chance, auf weiterführende Informationen zu verweisen und einfacher Daten einzuspeisen. Komplexere Inhalte, die eine App überfrachten würden, könnten untergebracht werden. Dazu gehören z. B. Videotutorials oder eine Linksammlung zu weiterführenden Themen im Bereich Versicherungen oder Finanzen. Sinnvoll wäre dabei auch eine Funktion zur Filterung von Informationen hinsichtlich der eigenen Relevanz (Kim et al. 2017). Da aktuelle Forschungsergebnisse aus Deutschland zeigen, dass besonders ältere Menschen ab 65 Jahren Probleme im Umgang mit digitalen Gesundheitsinformationen haben (Schaeffer et al. 2021), ist eine übersichtliche und ansprechende Aufbereitung der Information von zentraler Bedeutung. Wie aus den Interviews ersichtlich wurde, ist die zentrale Komponente der erfolgreichen Nutzung der App eine klare und intensive Einarbeitung. Videotutorials könnten hierzu auf der Website oder in den sozialen Medien platziert werden.

Ein vierter möglicher Erweiterungsbereich ist ein *Notfallpaket* mit integriertem Notfallmanagement, inklusive der Hinterlegung von Gesundheitsdaten und wichtigen Dokumenten der zu pflegenden Person zur Weiterleitung an eine:n medizinische:n Ansprechpartner:in. Wichtige zusätzliche Notfalltelefonnummern könnten hier ebenfalls abgespeichert werden. Vorbild könnte hier der Notfallbereich der App „Checkpoint C“ sein (Barsch 2019).

Die Integration der App in *andere therapeutische Behandlungen mit gemeinsamer Nutzung von Daten* ist ein fünfter möglicher Erweiterungsbereich. In der jungen Branche des Telemonitoring sind digitale Angebote für die Betreuung einer zu pflegenden Patient:innengruppe bisher noch ein kleines Segment. Diesem könnte durch die unkomplizierte Möglichkeit einer Datenweitervermittlung zu anderen Betreuungsinstanzen der zu pflegenden Person hier Vorstoß geleistet werden. Zudem könnte die Möglichkeit einer direkten Kommunikationsmöglichkeit mit medizinischem Fachpersonal überlegt werden (wie z. B. der Fall bei Slater et al. 2018).

Weiterhin, als 6. Bereich und explizit vorgeschlagen von einer Interviewteilnehmerin, könnte ein *eigener App-Bereich für die zu pflegenden Personen* generiert werden. Zwar liegen dazu schon zahlreiche assistierte Technologien vor (z. B. in Garay et al. 2019), jedoch könnte sich die App an dieser Stelle darauf beschränken, die zu pflegende Person für ihre eigenen Bedürfnisse zu sensibilisieren und, solange wie möglich, in der Selbstständigkeit zu halten.

Einige bereits vorhandene Apps fokussierten sich zusätzlich auf die emotionalen Bedürfnisse von Pflegenden Angehörigen. Dazu gehören z. B. die Bewertung des Wohlergehens der Pflegenden, eine Förderung der sozialen Beziehungen oder ein Forum, welches einen Austausch ermöglicht, wie in den USA (Wittenberg et al. 2019) oder in Deutschland (Rutz et al. 2019b) bereits entwickelt. Dieser Bereich wurde von den Interviewpartnerinnen dieser Studie nicht genannt. Wie eine aktuelle Review-Studie aus Spanien zeigt (Sala-González et al. 2021), ist es nicht die Quantität der Funktionen, die für die Nutzer:innen wichtig ist, sondern die Adressierung der konkreten Herausforderung, die typisch für die häusliche Pflege sind.

Eine Limitation dieser Studie ist der Selektionsbias. Da die Rekrutierung über den Newsletter im Netzwerk Demenz am besten funktionierte, ist in dieser Studie der Anteil von an Demenz erkrankten pflegebedürftigen Personen ohne hohe Notwendigkeit der Dokumentation medizinischer Werte außerordentlich hoch. Trotzdem versprachen sich fast alle Teilnehmerinnen einen Mehrwert durch die App. Jedoch gaben sie an, dieser Mehrwert würde mit zunehmender medizinischer Kontrolle der zu pflegenden Personen mit aller Wahrscheinlichkeit steigen. In einer nächsten Entwicklungsstufe sollte eine quantitative Evaluation mit breiterem Nutzer:innenspektrum erfolgen.

Eine Teilnehmerin der Studie war darüber hinaus jünger als die ursprünglich avisierte Zielgruppe, jedoch spiegelt dies auch die tatsächliche Versorgungsrealität mit einem großen Altersspektrum pflegender Angehöriger wider.

## Schlussfolgerungen

Die App Liv stößt aus Sicht pflegender Angehöriger auf eine relevante Versorgungslücke. Acht der 10 Interviewteilnehmerinnen gaben an, dass die App eine große Hilfe in ihrem Alltag darstellen würde. Eine Weiterentwicklung erscheint vor diesem Hintergrund lohnend.

Dazu müssten zunächst die oben genannten Weiterentwicklungsmöglichkeiten der App auf ihr Umsetzungspotenzial geprüft werden. Um den Prototyp bis zur Anwendungsreife zu bringen, empfehlen sich weitere Usability Tests, bei denen die funktionelle App direkt auf den Smartphones der Nutzer:innen getestet werden kann. Vor dem Hintergrund dieser Studie bietet sich eine Weiterentwicklung hauptsächlich in Richtung Personalisierung der App (nach Gesundheitszustand der zu pflegenden Person und dementsprechenden Anforderungen an die pflegenden Angehörigen) sowie Möglichkeiten der Prävention und digitalen Gesundheitsförderung durch die Ausarbeitung eines Informationsbereiches für pflegende Angehörige an.

Insgesamt zeigen die Ergebnisse der Studie, dass Apps für pflegende Angehörige insbesondere die Kommunikation zwischen den verschiedenen Pflegenden – sei es professionell oder als Angehörige – gezielt einbinden sollten.

## Footnotes

1 Die App Liv wurde von den Webdesignerinnen Camilla Scheplitz und Hannelore Braisch entwickelt.
